# PhytoPath: an integrative resource for plant pathogen genomics

**DOI:** 10.1093/nar/gkv1052

**Published:** 2015-10-17

**Authors:** Helder Pedro, Uma Maheswari, Martin Urban, Alistair George Irvine, Alayne Cuzick, Mark D. McDowall, Daniel M. Staines, Eugene Kulesha, Kim Elizabeth Hammond-Kosack, Paul Julian Kersey

**Affiliations:** 1 The European Molecular Biology Laboratory, The European Bioinformatics Institute, Hinxton, Cambridgeshire, CB10 1SD, UK; 2 Department of Plant Biology and Crop Science, Rothamsted Research, Harpenden, Herts, AL5 2JQ, UK; 3 Department of Computational and Systems Biology, Rothamsted Research, Harpenden, Herts, AL5 2JQ, UK

## Abstract

PhytoPath (www.phytopathdb.org) is a resource for genomic and phenotypic data from plant pathogen species, that integrates phenotypic data for genes from PHI-base, an expertly curated catalog of genes with experimentally verified pathogenicity, with the Ensembl tools for data visualization and analysis. The resource is focused on fungi, protists (oomycetes) and bacterial plant pathogens that have genomes that have been sequenced and annotated. Genes with associated PHI-base data can be easily identified across all plant pathogen species using a BioMart-based query tool and visualized in their genomic context on the Ensembl genome browser. The PhytoPath resource contains data for 135 genomic sequences from 87 plant pathogen species, and 1364 genes curated for their role in pathogenicity and as targets for chemical intervention. Support for community annotation of gene models is provided using the WebApollo online gene editor, and we are working with interested communities to improve reference annotation for selected species.

## INTRODUCTION

PhytoPath provides access to all sequenced and annotated plant pathogen genomes that have been submitted to the International Nucleotide Sequence Database Nucleotide Consortium (1) (GenBank (2), the European Nucleotide Archive (3) (ENA) and the DNA Databank of Japan (4)), and is updated approximately four times per year; see http://www.phytopathdb.org/pathogens_eg for a complete list. With each release, all newly submitted data are incorporated in the Ensembl Fungi, Protist and Bacterial portals (5); mapped to the annotations in the Pathogen-Host Interactions database (http://www.phi-base.org) (6); and incorporated into the PhytoPath search tool.

## A COMPLETE RESOURCE FOR ALL SEQUENCED PLANT PATHOGENS

The number of plant pathogen genomes in PhytoPath has increased from 14, when the PhytoPath resource was launched in January 2012, to a current total of 135, reflecting the increasingly widespread use of genome sequencing; the list includes the 10 most important fungal plant pathogens according to global scientific importance (7). Other pathogens included in the resource include several major current causes of concern to global food and feed security, for example, the Ug99 lineage of *Puccinia graminis* f. sp. *tritici* (a virulent agent of wheat stem rust in sub-Saharan Africa), the oomycete *Phytophthora infestans* (the causative agent of potato late blight disease) and various species and strains of *Fusarium*. The breakdown by kingdom is shown in Table 1.

**Table 1.** Total number of phytopathogenic genomes by kingdom

	Fungi	Protists	Bacteria	Total
Species	62	15	10	87
Genome sequences	88	23	24	135

Genomic data for each species are available through the Ensembl Genomes portals. Available data include: genome sequence, structural and functional annotation of protein coding and non-protein coding genes, variants and regulatory features and repeat features, DNA and peptide-based alignments and evolutionary histories for gene families. Access is provided via programmatic tools, including Perl and RESTful Application Program Interfaces, public MySQL database instances, FTP for bulk data downloads and an interactive genome browser. The browser gives a number of different views provided at various levels of resolution: the ‘Location’ view offers a bird-eye view of a specific region of the assembly; while gene, transcript and variant views focus on specific annotated features. Where the community has performed more than one gene build on the same reference genome, the alternative models can be visualized in a supplemental track. Currently we provide alternative gene sets for the fungi *Botrytis cinerea* (the causative agent of gray mold disease on numerous host species) and *Zymoseptoria tritici* (the causative agents of *Septoria tritici* blotch disease on wheat).

Genes are routinely annotated with InterPro (8) and the Gene Ontology (9), and the functional consequences of known sequence variants are predicted. Users can incorporate their own data for interpretation in the context of the reference data, either by uploading their data, or by accessing it remotely over HTTP or FTP, using common file formats such as BAM, GFF and VCF. Tools for data analysis include BLAST (10) and a ‘Variant Effect Predictor’ (11) that will interpret new sequence variations in terms of their predicted functional consequences for the existing gene models.

To support more complex queries the data are packaged into gene and variant-centric data warehouses, constructed using the BioMart (12) system which allow users to filter large data sets and to select attributes for display and download. Access to BioMarts is provided via a web-based query tool and through SOAP and RESTful web services.

## CURRENT DATA IN PHYTOPATH

### Comparative analysis

For each species, a representative genome is chosen, and all genes annotated on this reference are included in a protein-based comparative analysis, together with the genes of non-phytopathogenic relatives, which is especially useful when a related species has been studied in greater depth than the phytopathogen itself. Genes are first clustered according to sequence similarity, after which alignments are constructed and the ancestral history of the gene family (a ‘gene tree’) is deduced (13). Each tree is given a unique identifier and can be downloaded and published. These analyses are provided per kingdom (i.e. fungi, protists, bacteria); an additional analysis is available that shows the relationships between widely conserved genes from a more limited selection of species from across the taxonomic space. Six phytopathogenic species (*Zymoseptoria tritici*, *Puccinia graminis* f. sp. *tritici*, *Ustilago maydis*, *Phytophthora infestans, Ralstonia solanacearum and Xanthomonas campestris* p.v. *campestris*) have been included in this broad range analysis, alongside three other fungi, seven other protists and a total of 167 other genomes.

For closely related species, pairwise genomic alignments are computed using the tools lastZ (14) combined with the use of the UCSC chain/net algorithm (15). The available alignments are listed in Table 2. For pairs of species with assembled chromosomes, broader range data about large syntenic regions are also available.

**Table 2.** Whole genome alignments in PhytoPath

Clade	Aligned species (all against all)
*Hypocreales*	*Fusarium graminearum^a^*
	*Fusarium pseudograminearum*
	*Fusarium oxysporum^a^*
	*Fusarium fujikuroi^a^*
	*Fusarium solani^a^*
	*Fusarium verticillioides^a^*
	*Trichoderma virens*
	*Trichoderma reesei^b^*
*Pucciniales*	*Puccinia graminis* f. sp. *tritici* CRL 75-36-700-3
	*Puccinia graminis* f.sp*. tritici* Ug99
	*Puccinia triticina*
	*Melampsora larici-populina*
*Saccharomycetales*	*Ashbya gossipi^a^*
	*Saccharomyces cerevisiae^a,b^*
*Glomerellales*	*Colletotrichum graminicola*
	*Verticillium dahliae JR2^a^*
*Protists*	*Albugo laibachi*
	*Hyaloperonospora arabidopsidis*
	*Phytophthora infestans*
	*Phytophthora kernoviae*
	*Phytophthora lateralis*
	*Phytophthora parasitica*
	*Phytophthora ramorum*
	*Phytophthora sojae*
	*Pythium arrhenomanes*
	*Pythium aphanidermatum*
	*Pythium irregulare*
	*Pythium iwayamai*
	*Pythium ultimum*
	*Pythium vexans*
	(Plus 19 non-phytopathogenic species)

^a^Species with fully assembled chromosomes.

^b^Non-phytopathogenic species.

### Alignment to other sequence data

The Ensembl framework can display alignments to various sequencing-based experiments as tracks. These data can be used to validate current gene models or identify alternative isoforms and splice sites and also to indicate gene expression under different experimental conditions. Whenever there are data in dbEST (16) for a PhytoPath species, alignments are performed against the reference assembly using Exonerate (17) and incorporated into the database. EST alignments are available for the following species: *Fusarium graminearum* (18) *Fusarium verticillioides, Fusarium oxysporum, Fusarium solani, Phaeosphaeria nodorum, Puccinia triticina, Ustilago maydis, Magnaporthe oryzae, Leptosphaeria maculans, Melampsora larici-populina, Zymoseptoria tritici, Colletotrichum graminicola, Phytophthora infestans, Phytophthora sojae* and *Pythium ultimum*. High throughput sequencing data are also available: with each release, the ENA is scanned for available resequencing data, which is mapped to the genome using the Burrows-Wheeler Alignment Tool (19). Supplemental Table S1 gives a brief description of the available data set samples (20–24).

### Variation data sets

PhytoPath provides access to publicly available variant feature calls, including the location of variant loci, alleles and their distribution amongst individuals and populations, linkage disequilibrium, phenotypic associations resulting from whole genome analysis and their functional consequence on coding genes. For some data sets, however, sequence read data from resequencing experiments are present in the ENA, but the variant calls have not been published. Therefore, with each release of PhytoPath, such data are identified, mapped to the genome using the Burrows-Wheeler Alignment Tool (19), and variants are identified using SAMtools (25). In the future, we will additionally incorporate all variant calls submitted to the recently established European Variation Archive (26), which should provide a scalable model for accommodating an increased flow of new data. The complete set of variation data available is listed in Supplemental Table S2.

## LINKING GENOMES TO PHENOTYPIC DATA

PHI-base is a database of manual curated phenotypic data for genes that have been functionally investigated in pathogen-host studies (6,27). Version 3.8 contains more than 5000 interactions with over 60% involving plant-infecting pathogens. The integration of these data in Ensembl provides a way to view these data in their genomic context and perform complex analysis for the study of phytopathogens and the pathogenic process.

Where annotated genes have been identified in the context of a reference genome, mapping involves first picking up the taxon identifier from the PHI-base database, then activating the mapping process at that taxon level and finally searching for the matching UniProt accession in all the genomes belonging to that clade. However, many of the older annotations in PHI-base were derived from studies performed before a wide genome level annotation was performed in that species and are identified using a different database and/or some sequence. Other studies use a gene set that comes from a different gene build version, which can completely rename all genes with no mapping between both versions available in the public domain. In other cases, annotations in PHI-base have been associated with a species level pathogen while in Ensembl Genomes sequences may exist for several strains. Ambiguous mappings are completed using taxonomic identifiers, transitive cross-references to the UniProt Knowledgebase and direct sequence alignment. A summary of mapped alignments is provided in Table 3.

**Table 3.** Number of annotated interactions and individually annotated genes per species on PHI-base and Ensembl

	PHI-base genes	Interaction outcomes curated	Mapped genes on Ensembl
**Fungal species:**			
*Botrytis cinerea*	86	210	0
*Fusarium graminearum*	966	1078	864
*Fusarium oxysporum* f.sp. *lycopersici*	58	85	35
*Fusarium solani*	9	9	4
*Leptosphaeria maculans*	17	21	4
*Magnaporthe oryzae*	423	662	237
*Phaeosphaeria nodorum^a^*	40	46	24
*Sclerotina sclerotiorum*	9	22	2
*Ustilago maydis*	197	252	141
*Zymoseptoria tritici*	41	42	12
**Protist species:**			
*Hyaloperonospora arabidopsidis*	55	67	2
*Phytophthora infestans*	22	26	9
*Phytophthora sojae*	16	30	9
**Bacterial species:**			
*Burkholderia glumae*	14	23	5
*Erwinia amylovora*	20	33	0
*Pantoea stewartii*	6	6	0
*Pseudomonas syringae* pv *tomato*	8	12	4
*Pseudomonas syringae pv tabaci*	4	6	0
*Xanthomonas campestris* pv. *campestris*	5	5	1
*Xanthomonas oryzae* pv. *oryzae*	12	132	3

^a^Synonym Parastagonospora nodorum.

## DISPLAY OF AND ACCESS TO PHENOTYPIC DATA

**Figure 1.**
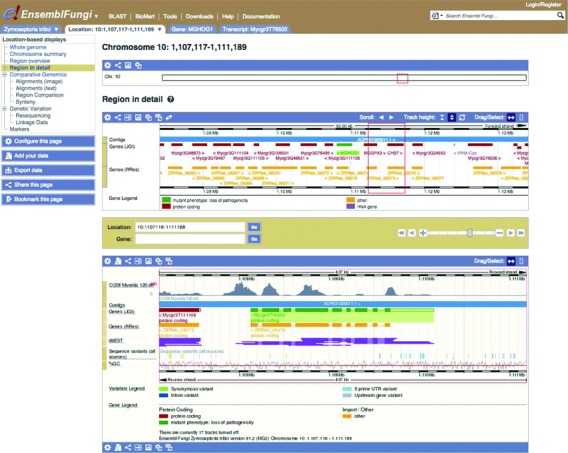
*Zymoseptoria tritici* region view in the Ensembl Genomes browser. Two gene build sets are represented in red and orange. The gene highlighted in green has a PHI-base annotation with a ‘loss of pathogenicity’ mutant phenotype. Expression data for mycelia are shown in the lower panel in gray alongside an EST track in purple and sequence variants represented by colored bands. The visibility of each track is chosen using the ‘Configure this page’ panel on the left.

PHI-base annotations contain information about the host, the experimental condition and the resulting phenotype. The link to the peer reviewed published article has also been imported. Phytopathogenic genes are highlighted in the browser, highlighting pathogenicity islands. If a gene has a single annotation or all the annotations confirm the same phenotype, it is highlighted with a color specific to the phenotype. A pop-up menu provides an immediate link to each of the PHI-base annotations associated with that gene, and a link to the gene page in PHI-base where additional information can be accessed through new design of the PHI-base website. These include details that may influence the observed interaction phenotype, such as the host strain, or chemistry that may act to induce or prevent a pathogenic outcome. Figure 1 shows a typical region view with one PHI-base annotated gene.

**Figure 2.**
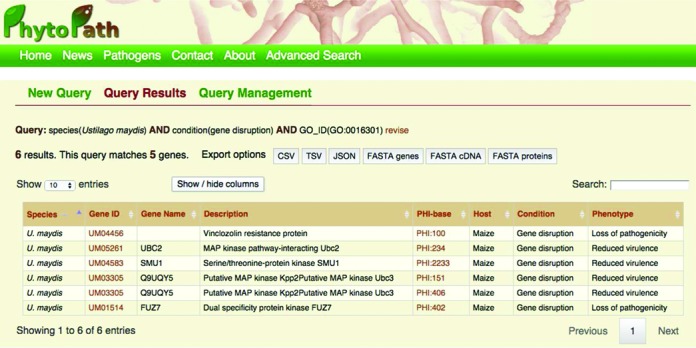
Typical results table displayed when using the PhytoPath advanced search.

All the PHI-base attributes stored in Ensembl are included in the BioMart (12) query tool. Ensembl BioMarts are typically constructed to provide access on a per-species basis, but through PhytoPath, a new interface is available supporting multi-species queries (http://www.phytopathdb.org/query/builder), and allowing users to navigate a taxonomic tree to select groups of related phytopathogens to query. Successive queries can be combined using set algebra and both gene annotation and sequences can be downloaded for further use. Figure 2 shows the results for a query consisting on the intersection of two filters, one regarding one PHI-base field and the other a GO annotation. There is the possibility to show GO data and protein domains.

## FUTURE DIRECTIONS

### Tools for community-led curation

We are working to support the community annotation of gene models. For interested communities, we can provide access to a private site, allowing groups to share data prior to publication and utilize the PhytoPath toolset while completing their annotation. In addition, we can provide access to the online editing tool WebApollo (28) for community members to directly submit new or modified gene models, which are immediately visible in public as an additional track and which are incorporated (following appropriate quality control) into the official gene set at periodic intervals. Groups seeking this support should contact us on http://www.phytopathdb.org/content/contact-us.

### Linking pathogen and host genomes

Pathogens and their hosts are directly engaged in communication and molecular warfare using secreted effector molecules and proteinaceous toxins. Both animal and plant pathogens aim to suppress early host detection by camouflage and to weaken the host's immune system once detected (29). Many pathogen delivered molecules have been shown to interact directly with one to a few extracellular or intracellular host target molecules. A current priority is to associate the host target genes in genomes available at Ensembl with the pathogen genes in PHI-base. The 231 pathogenic organisms in PHI-base have a total of 84 associated host species, of which eight plants are already available in Ensembl. Table 4 lists the host organisms associated with bacterial, fungal and protist pathogens in PHI-base Version 3.8.

**Table 4.** Host plants in PHI-base infected by two or more phytopathogenic species also in PHI-base

Host species	Pathogen type		
	Bacteria	Fungi	Protists
***Dicotyledonous plants***			
Arabidopsis thaliana*	2	8	2
*Brassica napus*	0	3	0
*Capsicum annuum*	4	1	1
*Cucumis sativus*	1	4	0
*Citrus sinensis*	1	2	0
*Glycine max*	2	2	1
*Lactuca sativa*	1	0	1
*Malus domestica*	1	2	0
*Nicotiana benthamiana*	2	2	3
*Nicotiana tabacum*	1	1	2
*Olea europaea*	1	0	0
*Phaseolus vulgaris*	2	4	0
*Pisum sativum*	1	2	0
*Pyrus communis*	0	3	0
Solanum lycopersicum*	5	11	3
Solanum tuberosum*	7	2	2
Vitis vinifera*	1	1	0
**Monocotyledonous plants**			
Hordeum vulgare*	0	9	0
Oryza sativa*	2	3	0
*Secale cereale*	0	3	0
Triticum aestivum*	0	10	0
Zea mays*	1	10	0

*Species present on Ensembl Plants.
